# Relationship between baseline bicarbonate and 30-day mortality in patients with non-traumatic subarachnoid hemorrhage

**DOI:** 10.3389/fneur.2023.1310327

**Published:** 2024-01-03

**Authors:** Wenyuan Du, Jingmian Yang, Yanfang Lou, Jiahua You, Qiang Wang

**Affiliations:** ^1^Department of Neurology, Shijiazhuang Traditional Chinese Medicine Hospital, Shijiazhuang, Hebei, China; ^2^Department of Cardiology, Shijiazhuang Traditional Chinese Medicine Hospital, Shijiazhuang, Hebei, China

**Keywords:** 30-day mortality, bicarbonate, non-traumatic subarachnoid hemorrhage, Intensive Care Unit, MIMIC-IV

## Abstract

**Objective:**

This study aimed to explore the relationship between baseline bicarbonate levels and 30-day mortality in individuals with non-traumatic subarachnoid hemorrhage (SAH).

**Methods:**

Patients with non-traumatic SAH were chosen from the Medical Information Mart for Intensive Care (MIMIC)-IV database. The relationship between baseline bicarbonate and 30-day mortality was examined using Cox regression models. Restricted cubic splines were used to test the hypothesis that there was an association between bicarbonate and mortality. With the use of Kaplan–Meier survival curve analysis, we looked deeper into the validity of these correlations. To find subgroups with differences, interaction tests were utilized.

**Results:**

This retrospective cohort study consisted of 521 participants in total. Bicarbonate had a negative association with death at 30 days (HR = 0.93, 95%CI: 0.88–0.98, *p* = 0.004). Next, we divided bicarbonate into quartile groups. In comparison to the reference group Q1 (20 mEq/L), groups Q3 (23–25 mEq/L) and Q4 (26 mEq/L) had adjusted HR values of 0.47 (95%CI: 0.27–0.82, *p* = 0.007) and 0.56 (95%CI: 0.31–0.99, *p* = 0.047). No definite conclusions can be derived from this study, since there is no obvious curve link between baseline bicarbonate and 30-day mortality. Patients’ 30-day mortality increased statistically significantly (*p* < 0.001, K–M analysis) in patients with low bicarbonate levels. The relationship between bicarbonate and 30-day mortality remained consistent in the stratified analysis, with no observed interactions.

**Conclusion:**

Finally, 30-day mortality was negatively associated with baseline bicarbonate levels. Patients with non-traumatic SAH are more at risk of mortality if their bicarbonate levels are low.

## 1 Introduction

A typical kind of stroke observed in intensive care units (ICUs) is non-traumatic SAH, which has an annual incidence rate of (7.2–9.0) per 100,000 individuals (1). Despite the improvement of comprehensive treatments, such as neuro-microsurgery, the case fatality rate among SAH patients is still very high (2). Even after survival, patients are still prone to residual neurological deficits, which seriously affect basic life.

The mechanism leading to secondary brain injury after SAH is multifactorial. Earlier research believed that post-SAH cerebral vasospasm was thought to be a major factor in the prognosis of the disease, but the clinical treatment of cerebral vasospasm did not significantly improve the neurological outcome of patients (3). In recent years, most experimental and clinical studies have focused on the pathophysiological mechanism of early brain injury (EBI), and it is believed that this stage may be closely related to poor outcomes of patients (4). EBI is defined as the pathological events in the first 72 h after SAH, which has become a research hotspot in the field of neurology (5). The pathological mechanism of EBI is relatively complex, and the potential mechanisms found so far include ischemic injury, oxidative stress response, neuroinflammation, cerebral microvascular dysfunction, blood–brain barrier destruction, apoptosis, and cortical diffuse depolarization. Based on the pathological mechanism of EBI, the study of reliable early biological markers can facilitate early prognosis assessment and treatment for SAH patients.

Early cerebral ischemic injury in SAH is an important prognostic factor. In a rat model of SAH, when cerebral blood flow (CBF) fell to 40% of baseline within 60 min after SAH, “lethal” SAH was induced (6). The decreased self-regulation ability of cerebral vessels after SAH is one of the main reasons for the decrease in CBF (7). Endothelial cells regulate vasodilation and contraction by producing cytokines and chemical transmitters, which are one of the pathological mechanisms of all clinical subtypes of stroke (8). There is proof that endothelial dysfunction and reduced serum bicarbonate levels may be related (9). In addition, changes in acid–base balance can directly affect CBF (10), and arterial bicarbonate is recognized as a biological marker for the clinical assessment of acid–base status. By producing free radicals, impairing glutamate absorption, activating glial cells, and inducing neuronal apoptosis, acidosis itself may adversely affect neurons and exacerbate ischemic brain injury (11). In previous studies, there was evidence that sodium bicarbonate can reduce intracranial pressure in patients with traumatic brain injury (12). Bicarbonate was included in predictive models as an important prognostic factor in SAH patients (13, 14). Other studies have shown that acute ischemic stroke patients are more likely to experience mortality when their baseline bicarbonate levels are low and their bicarbonate levels decrease while they are receiving critical care (15). There are currently no published clinical investigations on the relationship between bicarbonate and the prognosis of SAH patients. Therefore, it is important to understand the connection between bicarbonate and the rate of mortality in SAH patients.

We studied data from the MIMIC-IV database to figure out the relationship between bicarbonate levels and 30-day mortality among individuals with non-traumatic SAH.

## 2 Materials and methods

### 2.1 Data sources

The big public database MIMIC-IV (16) allows free downloads of the study’s data. The patients who were admitted to the Beth Israel Deaconess Medical Center (BIDMC) patients were listed in the database between 2008 and 2019. The first author was permitted to access the database (Record ID: 56305796) after passing the Protecting Human Research Participants exam and the National Institutes of Health (NIH) training course. Data collection does not need informed permission because all information is anonymized to safeguard patient privacy. The study adheres to the STROBE statement (17).

### 2.2 Study population

**Figure 1:**
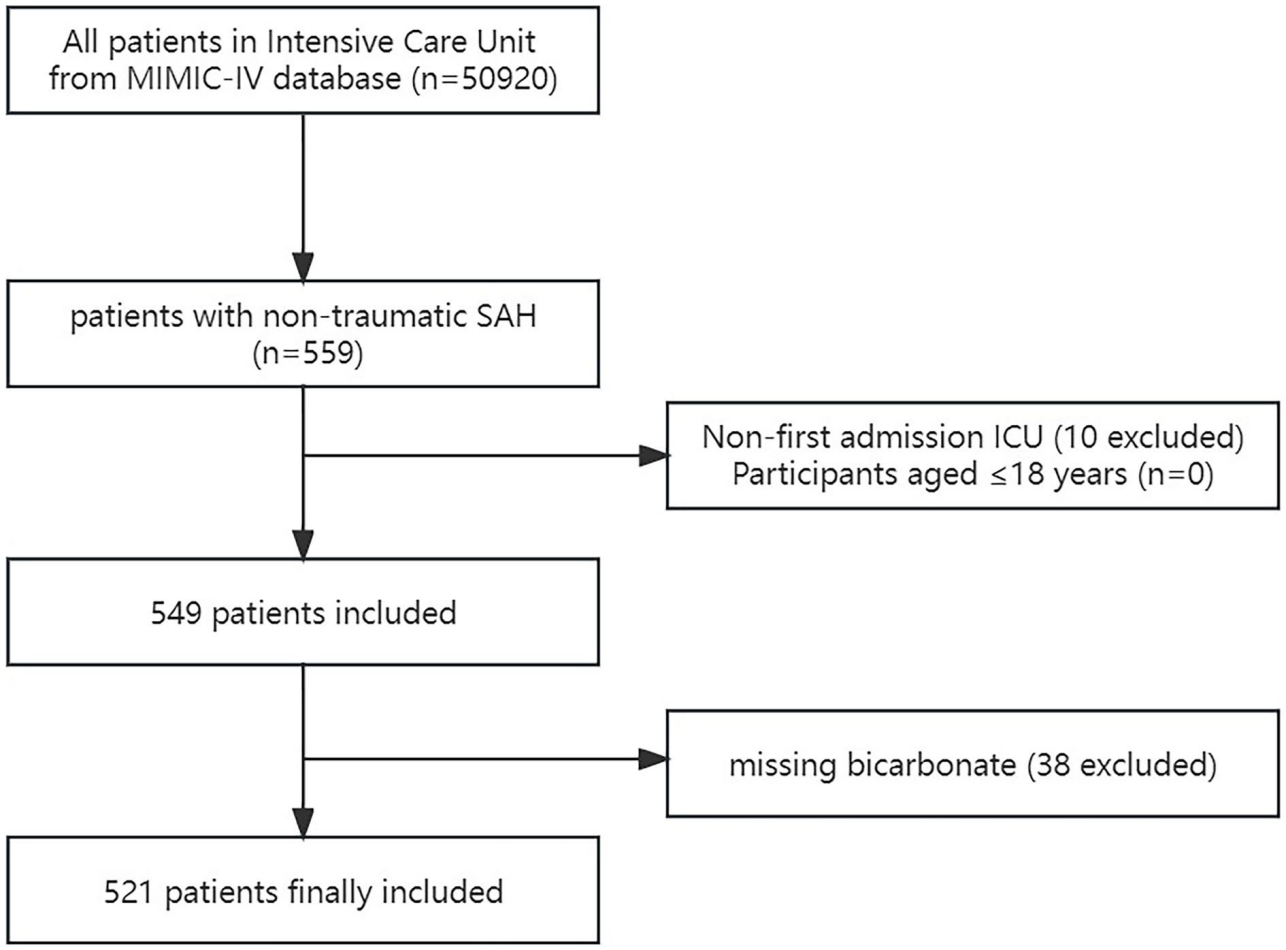
Flow chart of the study. MIMC-IV, Medical Information Mart for Intensive Care IV; ICU, intensive care unit; SAH, subarachnoid hemorrhage.

A total of 559 non-traumatic SAH patients were admitted to the ICU, according to ICD-9 code 430 and ICD-10 codes I60, I600-I609, I6000-I6002, I6010-I6012, I6020-I6022, I6030-I6032, and I6050-I6052 (18). The patients who satisfied the requirements were chosen for analysis: First ICU hospitalization and age older than 18 years. Participants missing bicarbonate readings were excluded. Finally, the research comprised 521 participants (Figure 1).

### 2.3 Data extraction

Using PostgreSQL, the following variables were retrieved for this study: (i) Sex, age, and race were among the demographic factors; (ii) respiratory rate (RR), heart rate, mean arterial blood pressure (MBP), blood oxygen saturation (SpO_2_), and mean temperature on the first day of ICU admission; (iii) The Charlson comorbidity index, chronic lung illness, paraplegia, hypertension, diabetes, sepsis, renal disease, cancer, severe liver disease, hydrocephalus, and congestive heart failure were among the complications; (iv) Laboratory variables included baseline blood glucose, platelets, white blood cell (WBC) count, red blood cells (RBCs), calcium, sodium, hemoglobin, PT, APTT, creatinine, urea nitrogen, and bicarbonate, tested for the first time within 24 h of admission to the ICU; (v) Baseline Glasgow Coma Score (GCS), Acute Physiology III (APSIII) score, SOFA score; and (vi) 30-day mortality, 24 h mortality, 48 h mortality, 7-day mortality, and in-hospital mortality (endpoints).

### 2.4 Statistical analysis

Continuous variables are represented using mean ± standard deviation (SD) or median (quartile 1, quartile 3). Depending on the distribution’s normality, the *t*-test or Mann–Whitney *U*-test is employed. The categorical variable is given as the percentage of instances, and the chi-square test (or Fisher’s exact method) is performed to examine the variations among the bicarbonate groups (grouped by quartile).

Cox proportional hazard regression analysis, both univariate and multivariate approaches, was employed. Based on the following criteria, confounding variables were screened: (i) the factor had a significant effect on the study variable (>10%); (ii) based on clinical experience and univariate analysis results, a few factors could have a significant effect on the result variables; and (iii) there are at least 10 valid sample sizes for each variable factor. To guarantee that the outcomes were consistent, we created three models. There were no adjustments made to the variables in the crude model. The variables such as age, sex, and race were adjusted in Model I, whereas in Model II, heart rate, respiratory rate (RR), hemoglobin, platelets, sepsis, Charlson comorbidity index, intravascular treatment, serum creatinine, and GCS score were all adjusted.

Using restricted cubic spline analysis, it was investigated whether there was a curve connection between baseline bicarbonate and 30-day mortality. The threshold effect of bicarbonate is determined using a two-piecewise linear regression model if a non-linear connection is observed. The linear model was contrasted using the log-likelihood ratio test as well (19). Interaction and stratified analyses were performed for age (<60 years and ≥ 60 years), sex, myocardial infarction, congestive heart failure, hypertension, diabetes, sepsis, endovascular aneurysm treatment, and GCS (<8 and ≥ 8 scores). A direct deletion is performed on the missing value. Supplementary Table S1 provides details about the missing values.

Statistical analysis was performed using packages R 3.3.2^1^ and free statistics software 1.8 (20, 21). Statistical significance was defined as a value of *p* of <0.05 (bilateral test).

## 3 Results

### 3.1 Baseline characteristics of the study patients

The final data analysis comprised 521 of the 559 non-traumatic SAH patients who matched the inclusion criteria and were first admitted to the ICU (Figure 1). The baseline bicarbonate level was divided into four groups according to the quartiles (Q1: ≤20 mEq/L; Q2: 21–22 mEq/L; Q3: 23–25 mEq/L; Q4: ≥26 mEq/L). The patients’ basic characteristics in each group are listed in Table 1. With a mean age of 60.2 years, the patients were mostly women (56%). Table 1 contains the patient’s demographic information, vital signs, comorbidity, hematological detection indicators, surgical treatment, score, outcome, and other relevant data. Significant distinctions were observed in sex, heart rate, RR, oxygen saturation, blood glucose, hemoglobin, platelet count, WBC, blood calcium, GCS score, and so on. Compared with Q1, Q2, and Q4, patients in Q3 had a lower risk of 30-day death.

**Table 1:** Population characteristics by quartiles of the baseline bicarbonate level.

Variables	Quartiles of bicarbonate					value of *p*
	Total (*n* = 521)	Q1 (≤20 mEq/L) (*n* = 87)	Q2 (21–22 mEq/L) (*n* = 107)	Q3 (23–25 mEq/L) (*n* = 187)	Q4 (≥26 mEq/L) (*n* = 140)	
Demographics						
Female, *n* (%)	292 (56.0)	62 (71.3)	65 (60.7)	104 (55.6)	61 (43.6)	< 0.001
Age, years	60.2 ± 14.4	58.7 ± 14.2	59.6 ± 15.3	61.6 ± 14.3	59.8 ± 13.9	0.407
Ethnicity, *n* (%)						0.529
White	326 (62.6)	52 (59.8)	62 (57.9)	120 (64.2)	92 (65.7)	
Black	46 (8.8)	5 (5.7)	10 (9.3)	16 (8.6)	15 (10.7)	
Asian	17 (3.3)	6 (6.9)	3 (2.8)	5 (2.7)	3 (2.1)	
Other	132 (25.3)	24 (27.6)	32 (29.9)	46 (24.6)	30 (21.4)	
Vital signs						
Heart rate, beats/min	78.4 ± 13.7	83.3 ± 14.5	78.9 ± 15.0	76.6 ± 12.4	77.3 ± 12.9	0.001
MBP, mmHg	81.9 ± 8.8	81.1 ± 9.5	82.5 ± 9.0	82.0 ± 8.6	81.6 ± 8.7	0.699
RR, times/min	18.0 ± 3.4	19.8 ± 4.0	18.3 ± 3.4	17.4 ± 2.7	17.6 ± 3.4	< 0.001
Temperature, ^°^C	37.0 ± 0.5	36.9 ± 0.8	37.0 ± 0.5	37.0 ± 0.4	37.0 ± 0.5	0.431
SpO_2_, %	97.8 (96.3, 99.0)	98.3 (96.3, 99.2)	98.0 (96.1, 99.1)	97.8 (96.6, 99.0)	97.3 (95.9, 98.6)	0.024
Comorbidities, *n* (%)						
Myocardial infarction	40 (7.7)	9 (10.3)	8 (7.5)	12 (6.4)	11 (7.9)	0.728
Congestive heart failure	39 (7.5)	8 (9.2)	9 (8.4)	10 (5.3)	12 (8.6)	0.578
Chronic pulmonary disease	77 (14.8)	12 (13.8)	10 (9.3)	30 (16)	25 (17.9)	0.276
Hypertension	260 (49.9)	39 (44.8)	50 (46.7)	93 (49.7)	78 (55.7)	0.359
Diabetes	70 (13.4)	13 (14.9)	17 (15.9)	18 (9.6)	22 (15.7)	0.298
Paraplegia	61 (11.7)	6 (6.9)	16 (15)	20 (10.7)	19 (13.6)	0.296
Sepsis	258 (49.5)	52 (59.8)	52 (48.6)	95 (50.8)	59 (42.1)	0.076
Renal disease	27 (5.2)	7 (8)	8 (7.5)	7 (3.7)	5 (3.6)	0.234
Malignant cancer	21 (4.0)	7 (8)	1 (0.9)	6 (3.2)	7 (5)	0.063
Severe liver disease	6 (1.2)	1 (1.1)	1 (0.9)	2 (1.1)	2 (1.4)	1
Hydrocephalus	154 (29.6)	25 (28.7)	33 (30.8)	63 (33.7)	33 (23.6)	0.255
Charlson comorbidity index	4.0 (3.0, 6.0)	4.0 (3.0, 6.0)	4.0 (3.0, 6.0)	4.0 (3.0, 6.0)	5.0 (3.0, 6.0)	0.440
Laboratory results						
Glucose, mg/dl	142.7 ± 36.5	160.8 ± 47.9	144.4 ± 35.4	136.6 ± 30.7	138.1 ± 32.8	< 0.001
RBC, 10^12^/L	3.9 ± 0.7	3.8 ± 0.8	4.0 ± 0.7	4.0 ± 0.6	4.0 ± 0.6	0.124
Hemoglobin, g/L	11.9 ± 1.9	11.3 ± 2.2	11.8 ± 2.1	12.1 ± 1.8	12.0 ± 1.8	0.012
Platelets, 10^9^/L	215.7 ± 88.6	192.0 ± 78.3	239.4 ± 101.8	212.8 ± 91.4	215.9 ± 75.5	0.003
WBC, 10^9^/L	11.1 ± 4.8	12.4 ± 5.9	11.4 ± 4.4	10.6 ± 4.6	10.7 ± 4.6	0.019
Sodium, mmol/L	138.0 ± 3.9	137.2 ± 3.5	138.0 ± 3.7	137.9 ± 3.8	138.6 ± 4.5	0.080
Calcium, mmol/L	8.3 ± 0.7	7.9 ± 0.7	8.4 ± 0.8	8.3 ± 0.6	8.5 ± 0.6	< 0.001
PT, s	12.2 (11.3, 13.0)	12.3 (11.3, 13.0)	12.1 (11.2, 12.7)	12.2 (11.3, 12.9)	12.3 (11.4, 13.5)	0.509
APTT, s	26.1 (23.6, 28.5)	25.5 (23.6, 28.5)	26.4 (23.3, 28.5)	26.3 (24.1, 28.8)	25.8 (23.4, 28.3)	0.641
Cr (mg/dL)	0.7 (0.6, 0.9)	0.7 (0.6, 1.0)	0.7 (0.6, 1.0)	0.7 (0.6, 0.9)	0.8 (0.6, 1.0)	0.352
BUN (mg/dL)	12.0 (10.0, 17.0)	12.0 (9.0, 17.0)	11.0 (9.0, 16.0)	13.0 (10.0, 15.5)	13.0 (10.0, 18.0)	0.251
Therapy, *n* (%)						
Endovascular therapy	190 (36.5)	36 (41.4)	38 (35.5)	74 (39.6)	42 (30)	0.235
Clipping of aneurysm	37 (7.1)	8 (9.2)	10 (9.3)	14 (7.5)	5 (3.6)	0.253
**Scores**						
GCS	12.0 (7.0, 14.0)	9.0 (5.0, 14.0)	12.0 (6.0, 14.0)	13.0 (7.0, 14.0)	13.0 (8.0, 14.2)	0.030
APSIII	39.0 (27.0, 61.0)	59.0 (36.0, 77.0)	43.0 (28.0, 62.5)	35.0 (27.0, 52.5)	35.0 (25.8, 56.5)	< 0.001
SOFA	3.0 (2.0, 4.0)	3.0 (2.0, 5.0)	2.0 (2.0, 3.0)	2.0 (2.0, 3.0)	2.0 (2.0, 4.0)	0.003
Outcomes						
30-day mortality, *n* (%)	123 (23.6)	35 (40.2)	34 (31.8)	30 (16)	24 (17.1)	< 0.001

MBP, mean blood pressure; RR, respiratory rate; SpO2, percutaneous oxygen saturation; RBC, red blood cell; WBC, white blood cell; PT, prothrombin time; APTT, activated partial thromboplastin time; Cr, creatinine; BUN, Blood urea nitrogen; GCS, Glasgow coma score; APSIII score, Acute Physiology III score; SOFA, Sequential Organ Failure Assessment.

The non-survival group had lower bicarbonate levels (22.1 ± 4.0 vs. 23.8 ± 3.2) compared with the survival group. In addition, the non-survival group had the characteristics of older age (66.2 ± 14.5 vs. 58.4 ± 13.9), a higher incidence of comorbidity, such as chronic lung disease, sepsis, kidney disease and a lower GCS score, as shown in Supplementary Table S2.

### 3.2 Association of baseline bicarbonate with 30-day mortality

**Figure 2:**
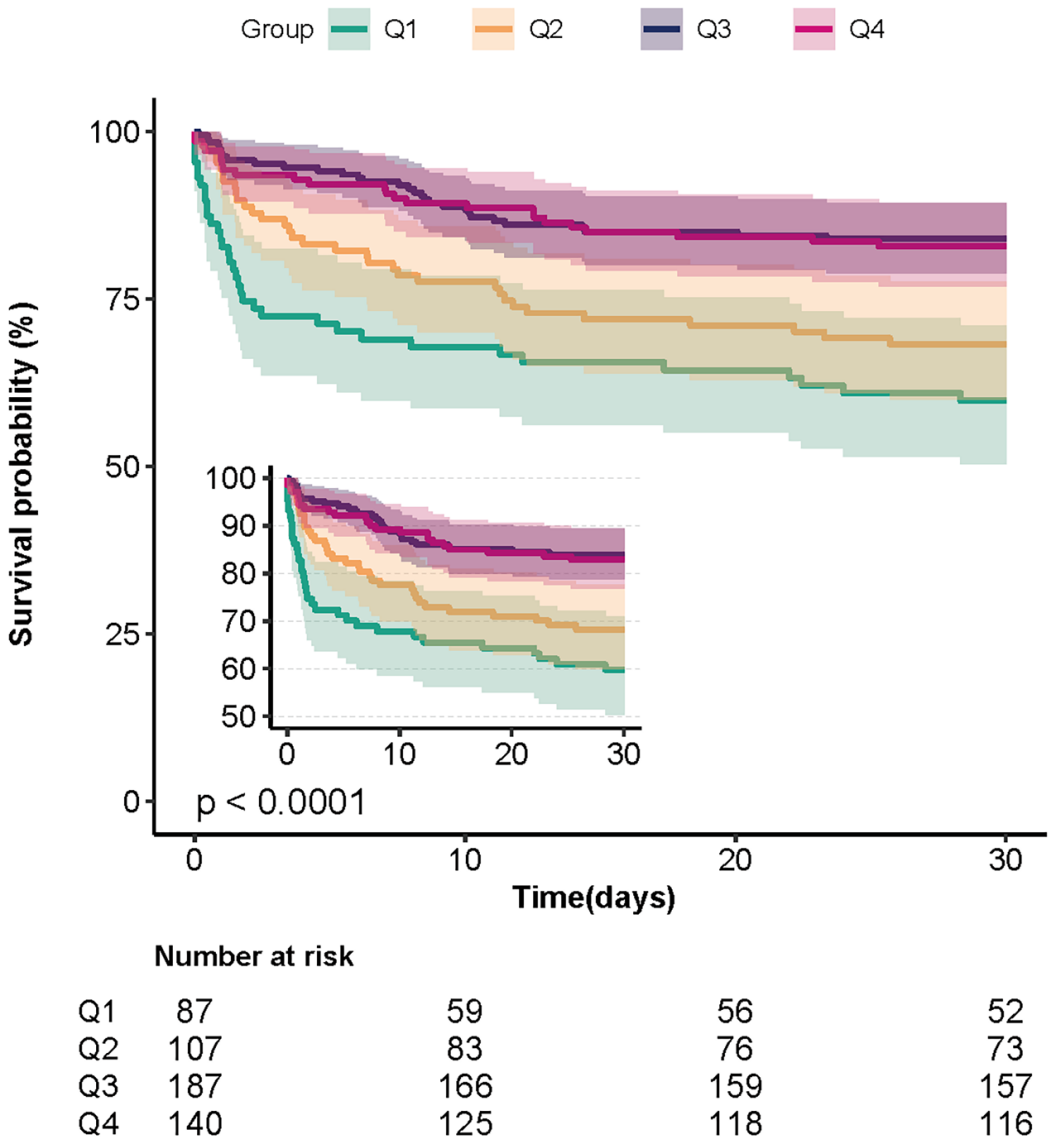
Kaplan–Meier survival curves for patients with non-traumatic SAH based on the baseline bicarbonate level. X-Axis: survival time (days). Y-Axis: survival probability. SAH, subarachnoid hemorrhage.

Univariate analysis showed that, in addition to baseline bicarbonate, age, RR, heart rate, paraplegia, sepsis, severe liver disease, Charlson comorbidity index, hemoglobin, platelet count, WBC, calcium, whether endovascular therapy was performed, and GCS score was associated with 30-day mortality (Supplementary Table S3). In Table 2, both the unadjusted and multivariate-adjusted relationships between baseline bicarbonate and 30-day mortality were observed. Model 1 was adjusted for age, sex, and race. Based on Model I, 12 variables, including heart rate, RR, sepsis, Charlson comorbidity index, hemoglobin, platelet count, creatinine, endovascular therapy, and GCS score, were further adjusted in Model II. Bicarbonate was linked to 30-day mortality according to the findings (unadjusted model: HR = 0.88, 95%CI: 0.83–0.92, *p* < 0.001; Model II: HR = 0.93, 95%CI: 0.88–0.98, *p* = 0.004). Results from Model II suggest that 30-day mortality is reduced by 7% for every 1 mEq/L increase in bicarbonate. When bicarbonate was used as a categoric variable, compared with the reference group, Q1 (bicarbonate≤20 mEq/L), the adjusted HR values of Q3 (23–25 mEq/L) and Q4 (≥26 mEq/L) groups were 0.47 (95%CI: 0.27–0.82, *p* < 0.001) and 0.56 (95%CI: 0.31–0.99, *p* < 0.001). The K–M curves between the four groups are shown in Figure 2. Multivariate regression analyses of baseline bicarbonate levels with 24 h, 48 h, 7-day mortality, and in-hospital mortality were also performed, and the results remained stable (Supplementary Table S4). The K–M curves for 24 h, 48 h, 7-day survival rates, and in-hospital survival rates are shown in Supplementary Figure S1.

**Table 2:** Multivariate Cox regression analyses for 30-day mortality in non-traumatic subarachnoid hemorrhage patients.

Exposure	Non-adjust model		Model I		Model II	
	HR (95% CI)	Value of *p*	HR (95% CI)	Value of *p*	HR (95% CI)	Value of *p*
Bicarbonate quartiles						
Q1 (≤20)	1 (Ref)		1 (Ref)		1 (Ref)	
Q2 (21–22)	0.7 (0.44, 1.12)	0.138	0.68 (0.42, 1.1)	0.119	0.94 (0.56, 1.59)	0.82
Q3 (23–25)	0.32 (0.2, 0.52)	<0.001	0.29 (0.18, 0.48)	<0.001	0.47 (0.27, 0.82)	0.007
Q4 (≥26)	0.35 (0.21, 0.58)	<0.001	0.35 (0.21, 0.6)	<0.001	0.56 (0.31, 0.99)	0.047
*p* for trend	0.66 (0.55, 0.78)	<0.001	0.65 (0.54, 0.77)	<0.001	0.77 (0.64, 0.93)	0.007
Bicarbonate (per 1 increases)	0.88 (0.83, 0.92)	<0.001	0.87 (0.83, 0.92)	<0.001	0.93 (0.88, 0.98)	0.004

Non-adjusted: no covariates were adjusted. Model I: adjusted for age, sex, and ethnicity. Model II: adjusted for age, sex, ethnicity, heart rate, RR, sepsis, hemoglobin, platelets, Charlson comorbidity index, Cr, endovascular therapy and GCS. RR, respiratory rate; Cr, creatinine; GCS, Glasgow coma score; HR, hazard ratio; CI, confidence interval; Ref, reference.

### 3.3 Association of baseline bicarbonate with 30-day mortality

An L-shaped connection between bicarbonate and 30-day mortality was found using multivariate-adjusted restricted cubic spline analysis with variables from Model I (Supplementary Figure S2, *p* = 0.017). We discovered that the bicarbonate threshold was 25.67 mEq/L using a two-piecewise linear model (Supplementary Table S5). The HR value was 0.83 (*p* < 0.001) on the left side of the threshold (95% CI: 0.78–0.88). This suggests that the probability of mortality dropped by 17% for every 1 mEq/L increase in bicarbonate. The HR value was 1.02 (95% CI: 0.80–1.30, *p* = 0.85) on the right side of the threshold. It was suggested that, when the bicarbonate level was greater than 25.67 mEq/L, the correlation between bicarbonate and mortality was not statistically significant. This indicates that the 30-day risk of death no longer decreases with the increase in bicarbonate. A restricted cubic spline analysis using the covariates of Model II did not show a curve association between bicarbonate and mortality (*p* = 0.466, Supplementary Figure S3).

### 3.4 Subgroup analysis

**Figure 3:**
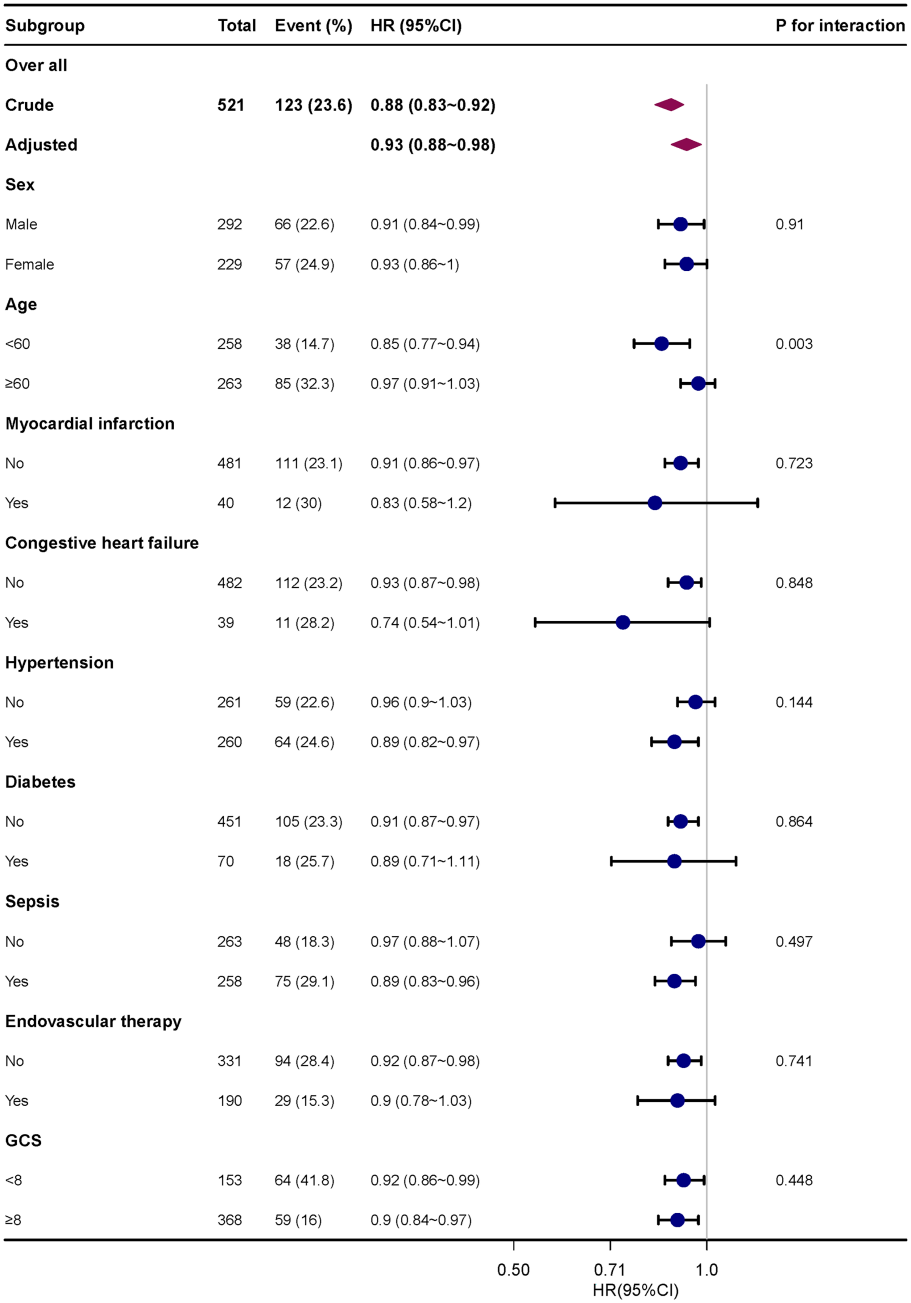
Subgroup analyses of the effect of 30-day mortality. Adjusted for age, sex, race, heart rate, RR, hemoglobin, platelets, sepsis, Charlson comorbidity index, intravascular therapy, serum creatinine and GCS score. RR, respiratory rate; GCS, Glasgow coma score.

Subgroup analysis revealed no significant interactions among different subgroups, including age (<60 years and ≥ 60 years), sex, myocardial infarction, congestive heart failure, hypertension, diabetes, sepsis, endovascular treatment, and GCS (<8 and ≥ 8) (Figure 3). The 30-day mortality rate and bicarbonate had a consistent relationship.

## 4 Discussion

We studied the connection between initial bicarbonate levels and 30-day mortality in non-traumatic SAH patients. Our analysis showed that, after accounting for other factors, bicarbonate had a negative correlation with 30-day mortality. This information can be beneficial in predicting outcomes for individuals with non-traumatic SAH who are admitted to the ICU.

Bicarbonate is an important component in regulating the acid–base balance of the human body, and the imbalance of acid–base balance is closely related to the outcome of critical patients (22–28). SAH is a common type of stroke in the ICU and has a high mortality rate. Recent research has revealed a strong correlation between EBI and a bad prognosis in SAH patients (4). After SAH, there will be several alterations in cerebral perfusion. Rapid blood extravasation causes a significant rise in intracranial pressure and a steady decline in cerebral perfusion pressure and CBF. The decrease in cerebral perfusion pressure leads to cerebral ischemia and a series of functional and metabolic disorders, which in turn increase the permeability of cell membranes to ions and lead to brain cell edema. The occurrence of brain edema further promotes the increase of intracranial pressure, the decrease of CBP, and the injury of endothelial cells, and then cerebral vasospasm occurs in the acute stage of cerebral hemorrhage, forming a vicious cycle and aggravating cerebral ischemic injury (7). Changes in acid–base balance can directly affect CBF (10), and acidosis can also exacerbate ischemic brain injury by causing endothelial inflammatory gene expression, producing free radicals, influencing glutamate reuptake, stimulating glial cells, and inducing neuronal apoptosis (11, 29–31). Dysfunction of vascular endothelium and vascular glial cells destroys the blood–brain barrier, allowing various substances in the blood to exudate into the brain parenchyma through the blood vessel wall, causing hydrocephalus, and aggravating the pathological process of EBI in SAH patients (32). Low serum bicarbonate levels indicate metabolic acidosis or a potential metabolic acidosis state. Animal experiments show that, in the rat model of permanent middle cerebral artery occlusion, the low bicarbonate group has a higher mortality rate (33, 34). Higher cerebrospinal fluid pH has been linked in clinical investigations to the emergence of delayed cerebral ischemia following SAH (35). Astrocytic dysfunction, which is adversely correlated with outcomes in stroke patients, may result from low bicarbonate levels (36). In previous studies, bicarbonate had been included in several stroke prognosis models (13, 14, 37). Bicarbonate is produced by carbonic anhydrase. The use of sodium bicarbonate was linked to a considerably decreased incidence of severe cardiovascular adverse events and mortality in individuals with advanced chronic renal disease (38, 39). Numerous investigations conducted recently suggest that carbonic anhydrase inhibitors may represent a novel therapeutic option for cerebral ischemia (13, 40). According to the results of our study, individuals with SAH had a higher 30-day risk of mortality when their baseline bicarbonate levels were low. We hypothesize that the increased mortality in SAH patients due to low bicarbonate levels may include the following reasons: Low bicarbonate levels indicate metabolic acidosis or potential metabolic acidosis. On the one hand, acidosis aggravates the pathological process of EBI in SAH patients by reducing cerebral blood flow, causing endothelial cell dysfunction, *and otherwise* (as described above). On the other hand, metabolic acidosis can have a range of harmful effects on the body. It can compromise the heart muscle’s contractility, heighten the likelihood of heart arrhythmias, diminish the tone of blood vessels throughout the body, reduce the body’s response to stress hormones, and cause pulmonary blood vessels to constrict. Furthermore, metabolic acidosis can impair the immune system and hamper white blood cell function, increasing the risk of infections for patients (41). These findings highlight the significance of bicarbonate monitoring in SAH patients and recommend that doctors offer targeted therapies to patients who have lower bicarbonate levels.

No statistical difference was found in mortality risk between group Q1 (≤20 mEq/L) and group Q2 (21–22 mEq/L) when bicarbonate was categorized by quartiles. In groups Q3 (23–25 mEq/L) and Q4 (≥26 mEq/L), the risk of mortality was reduced by 53 and 44%, respectively, in comparison to Q1. We thus attempted to observe whether there was a curve association between bicarbonate and 30-day mortality. An L-shaped connection between bicarbonate and 30-day mortality was demonstrated in Model I (*p* = 0.017). The threshold was discovered to be 25.67 mEq/L. The HR value was 0.83 (*p* = 0.001) on the left side of the threshold (95% CI: 0.78–0.88). This suggests that the probability of mortality dropped by 17% for every unit higher for bicarbonate. The HR value was 1.02 (95% CI: 0.80–1.30, *p* = 0.85) on the right side of the threshold. It was claimed that the link between bicarbonate and 30-day mortality was not meaningful when the bicarbonate level was higher than 25.67 mEq/L. This indicates that the probability of mortality within 30 days no longer decreases when bicarbonate levels rise. Bicarbonate and 30-day mortality did not exhibit a significant curve connection in restricted cubic spline analysis with covariates from Model II (*p* = 0.466), indicating a progressive drop in 30-day mortality with increasing bicarbonate levels. To determine whether there is a curvilinear association between baseline bicarbonate and 30-day mortality, further research is required.

In this study, we included a total of 521 patients, including 190 patients (36.5%) who underwent endovascular therapy and 37 patients (7.1%) who underwent clipping surgery. This is generally consistent with the ratio reported in previously published studies of non-traumatic SAH using the MIMIC-IV database. The proportion of endovascular therapy and clipping surgery reported by Jiuling Liu et al. was 29.1 and 5.2%, respectively (42). Intracranial aneurysmal SAH is the most common cause of non-traumatic SAH. Other causes include perimesencephalic SAH, brain arteriovenous malformation, moyamoya disease, dural arteriovenous fistulas, vasculitis, intracranial venous system thrombosis, connective tissue diseases, intracranial tumors, complications of anticoagulation therapy, etiology unknown, and so on. In addition to medication for non-traumatic SAH, different surgical treatments are employed according to different etiologies. The main surgical methods for aneurysms include endovascular therapy and clipping, and for brain arteriovenous malformation, the methods include surgical resection and stereotactic radiotherapy (43). Although we have obtained the surgical treatment information of the patients to the best of our knowledge, we could not obtain further details on the etiological distribution and treatment regimen due to the limitations of the MIMIC-IV database. The possible reasons for only a low proportion of patients undergoing surgical treatment in this study include, but are not limited to, the other etiologies, unknown etiology, absence of indications for surgery, and death before surgery. pH and lactic acid were not included in the multifactor analysis because of their co-linearity with bicarbonate. Since many indicators of vital signs, comorbidities, and laboratory results are included in APSIII and SOFA scores, to avoid multicollinearity, APSIII and SOFA scores are not considered to be included in the adjustment model. In addition to sex, age, and race, we preferentially selected indicators with significant statistical differences in univariate regression and a clear influence on patient prognosis in clinical practice, such as sepsis, Charlson comorbidity index, creatinine, and endovascular therapy as adjustment variables.

Our study has the following advantages: (i) To date, the relationship between baseline bicarbonate and 30-day mortality in individuals with non-traumatic SAH has not been reported in published articles. (ii) This study uses data from the real world to create a larger, more ethnically diversified population study. Lower null values for bicarbonate and covariates included in multivariate regression analysis may reduce the selection bias. (iii) According to the results of our study, SAH patients who had low baseline bicarbonate levels had a lower chance of surviving within 30 days. These findings highlight the significance of bicarbonate monitoring in SAH patients, which may aid doctors in identifying individuals at high risk for non-traumatic SAH and point to the need for particular therapies for patients whose bicarbonate levels are dropping. (iv) The findings remained reliable when we additionally examined the other endpoints, including 24 h, 48 h, 7-day, and in-hospital mortality.

This study does have certain restrictions, though. First, retrospective observational designs inevitably have bias and confounding factors. Second, the results’ applicability to other groups of people may be constrained due to the use of single-center sample. As a result, caution must be exercised when interpreting and using our findings in different situations. Third, because of limitations in the MIMIC database, missing variables that might have had an impact on the model, including cerebral vasospasm, delayed cerebral ischemia, and intracranial hypertension, were not acquired. Fourth, since we only looked at the initial batch of bicarbonate data gathered during an ICU stay, our analysis was only able to provide information on a short-term bicarbonate assessment. We only gathered data for 30 days; therefore, we could only evaluate results over the short term. More research is needed to assess the long-term consequences. Fifth, grouping bicarbonate by quartiles was found to have significant intergroup differences; however, this study did not draw a definitive conclusion on whether there is a curve-like relationship between bicarbonate and 30-day mortality, and follow-up studies are needed to continue exploring this aspect. Sixth, in future studies, we aim to further explore the effect of trends in bicarbonate levels on prognosis in patients with non-traumatic SAH, as there are limitations in examining the effect of a single value of a biomarker measured once on clinical prognosis. However, low bicarbonate levels and 30-day mortality were revealed to be correlated.

## 5 Conclusion

Patients with low baseline bicarbonate levels should thus get greater consideration since they may have a higher 30-day mortality rate. This will allow clinicians to make better clinical decisions.

## Data availability statement

The data analyzed in this study was obtained from the Medical Information Mart for Intensive Care IV (MIMIC-IV) database, the following licenses/restrictions apply: to access the files, users must fulfill be credentialed users, complete the required training (CITI Data or Specimens Only Research) and sign the data use agreement for the project. Requests to access these datasets should be directed to PhysioNet, https://physionet.org/, DOI: 10.13026/6mm1-ek67.

## Ethics statement

The studies involving humans were approved by the Massachusetts Institute of Technology and Beth Israel Deaconess Medical Center. The studies were conducted in accordance with the local legislation and institutional requirements. The ethics committee/institutional review board waived the requirement of written informed consent for participation from the participants or the participants’ legal guardians/next of kin because patients’ informed permission was not necessary for this study since health information was anonymized.

## Author contributions

WD: Data curation, Methodology, Project administration, Software, Supervision, Writing – original draft, Writing – review & editing. JYa: Data curation, Writing – review & editing. YL: Writing – review & editing. JYo: Writing – review & editing. QW: Supervision, Writing – review & editing.

## Footnotes

^1^ http://www.R-project.org

## Supplementary material

SUPPLEMENTARY FIGURE S1 Kaplan–Meier survival curves for patients with non-traumatic SAH based on the baseline bicarbonate level. **(A)** shows the 24-hour survival probability from different bicarbonate groups. **(B)** shows the 48-hour survival probability from different bicarbonate groups. **(C)** shows the 7-day survival probability from different bicarbonate groups. **(D)** shows the in-hospital survival probability from different bicarbonate groups. X-Axis: survival time (hours/days). Y-Axis: survival probability. SAH, subarachnoid hemorrhage.

SUPPLEMENTARY FIGURE S2 Restricted cubic spline plot for association bicarbonate and 30-day mortality risk. The data were adjusted for the variables in Model I.

SUPPLEMENTARY FIGURE S3 Restricted cubic spline plot for association bicarbonate and 30-day mortality risk. The data were adjusted for the variables in Model II.
