# Supplementary figures and images for: Relationship between baseline bicarbonate and 30-day mortality in patients with non-traumatic subarachnoid hemorrhage
Source: Front Neurol. 2024 Jan 3;14:1310327. doi: 10.3389/fneur.2023.1310327 (PMC10793108; doi:10.3389/fneur.2023.1310327)

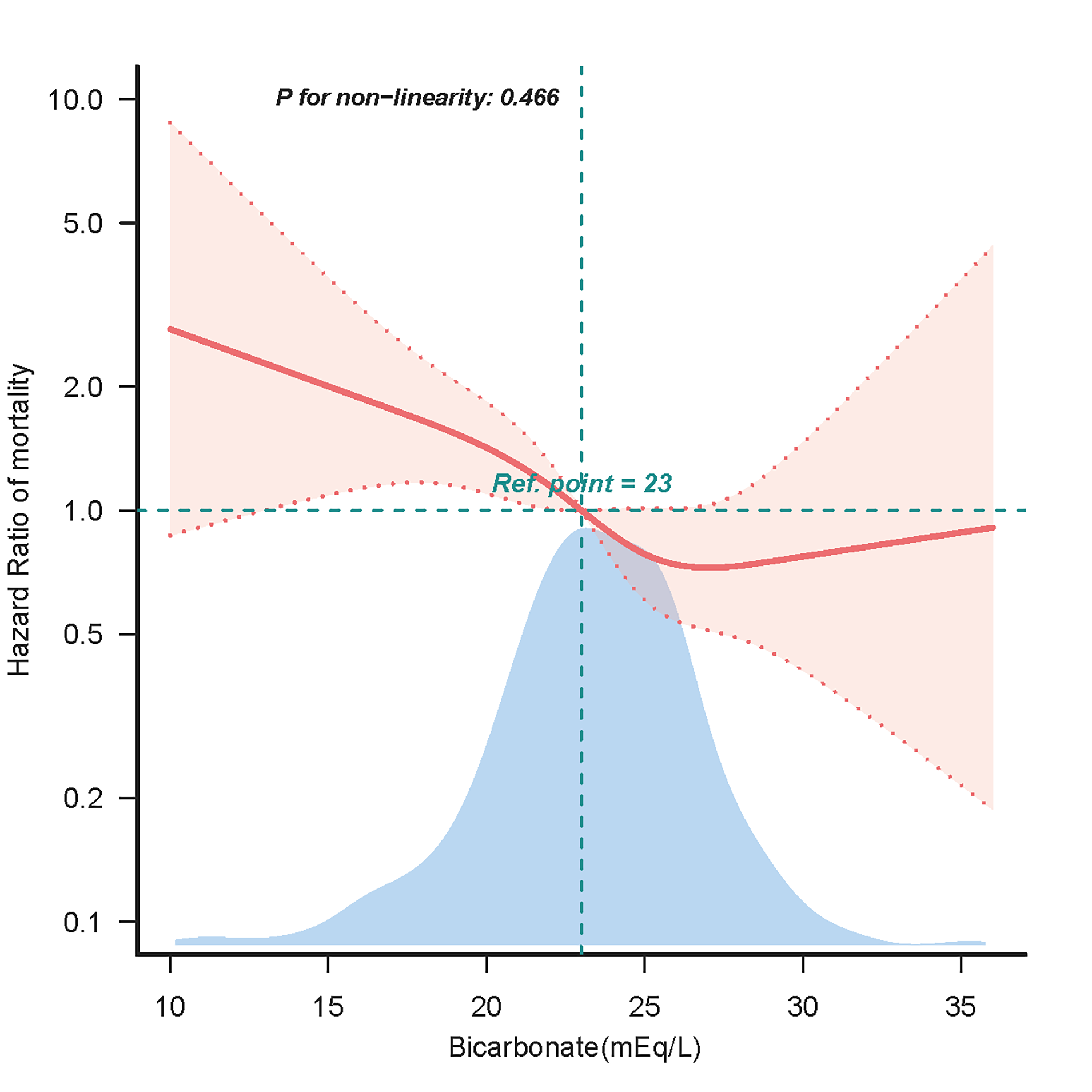

Supplement: SUPPLEMENTARY FIGURE S2 — Restricted cubic spline plot for association bicarbonate and 30-day mortality risk. The data were adjusted for the variables in Model I. [file Image_3.TIF]

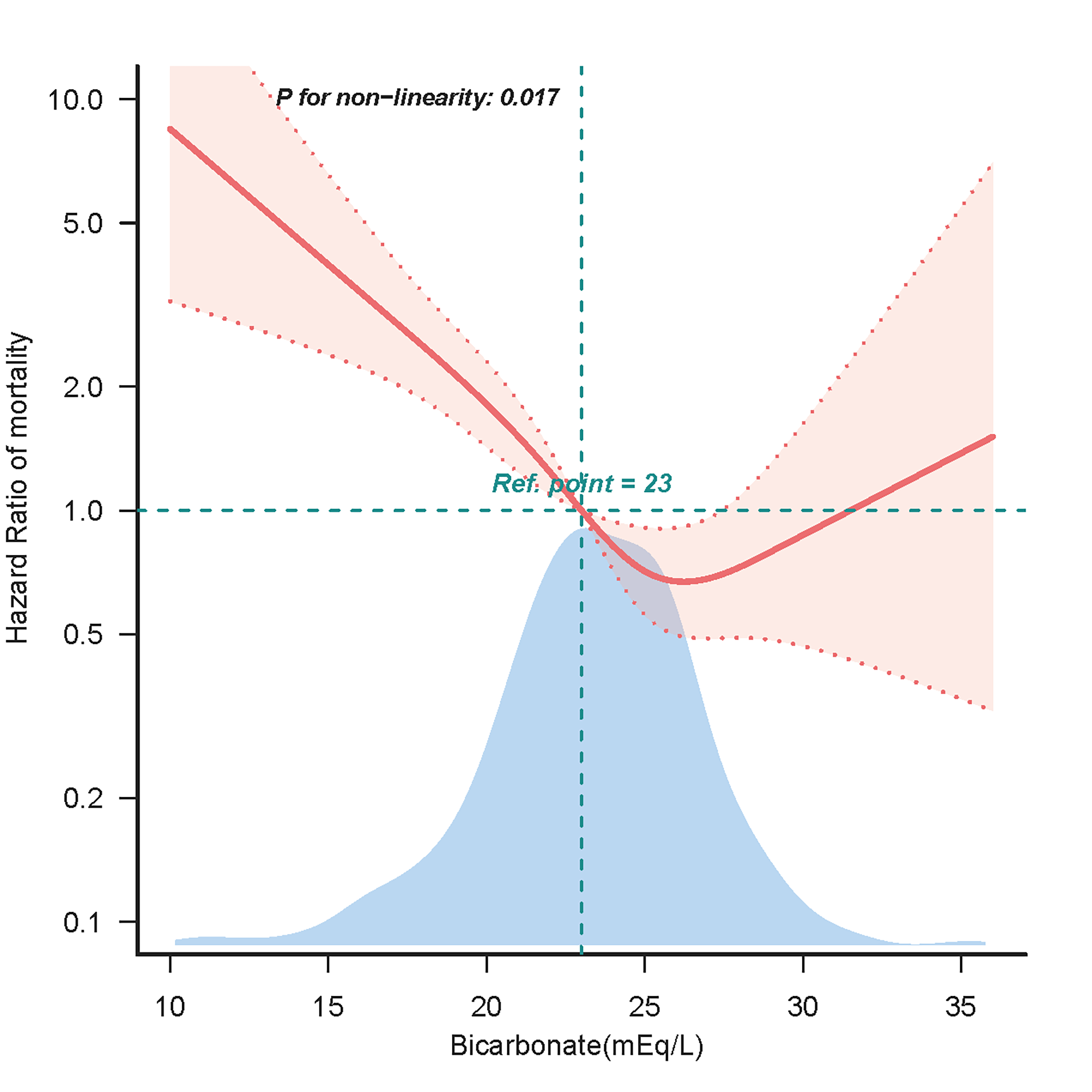

Supplement: SUPPLEMENTARY FIGURE S3 — Restricted cubic spline plot for association bicarbonate and 30-day mortality risk. The data were adjusted for the variables in Model II. [file Image_4.TIFF]
