# Supplementary material for: Relationship between baseline bicarbonate and 30-day mortality in patients with non-traumatic subarachnoid hemorrhage
Source: Front Neurol. 2024 Jan 3;14:1310327. doi: 10.3389/fneur.2023.1310327 (PMC10793108; doi:10.3389/fneur.2023.1310327)
Supplement: Supplementary file 4 [file Table_1.DOCX]

**Supplementary T1 Details of missing values**

| **Variable** | **Miss.freq** | **Miss.percentage%** |
| --- | --- | --- |
| Female | 0 | 0 |
| Age | 0 | 0 |
| Ethnicity | 0 | 0 |
| Heart rate | 1 | 0.1919 |
| MBP | 1 | 0.1919 |
| RR | 1 | 0.1919 |
| Temperature | 6 | 1.1516 |
| SpO_2_ | 1 | 0.1919 |
| Myocardial infarction | 0 | 0 |
| Congestive heart failure | 0 | 0 |
| Chronic pulmonary disease | 0 | 0 |
| Hypertension | 0 | 0 |
| Diabetes | 0 | 0 |
| Paraplegia | 0 | 0 |
| Sepsis | 0 | 0 |
| Renal disease | 0 | 0 |
| Malignant cancer | 0 | 0 |
| Severe liver disease | 0 | 0 |
| Charlson comorbidity index | 0 | 0 |
| Glucose | 1 | 0.1919 |
| RBC | 3 | 0.5758 |
| Hemoglobin | 0 | 0 |
| Platelets | 1 | 0.1919 |
| WBC | 0 | 0 |
| Sodium | 0 | 0 |
| Calcium | 8 | 1.5355 |
| PT | 7 | 1.3436 |
| APTT | 12 | 2.3033 |
| Cr | 0 | 0 |
| BUN | 0 | 0 |
| Endovascular therapy | 0 | 0 |
| Clipping of aneurysm | 0 | 0 |
| GCS | 0 | 0 |
| APSIII | 0 | 0 |
| SOFA | 263 | 50.4798 |
| Hydrocephalus | 0 | 0 |
| 30-day mortality | 0 | 0 |

MBP, mean blood pressure; RR, respiratory rate; SpO2, percutaneous oxygen saturation; RBC, red blood cell; WBC, white blood cell; PT, prothrombin time; APTT, activated partial thromboplastin time; Cr, Creatinine; BUN, Blood urea nitrogen; GCS, Glasgow coma score; APSIII score, Acute Physiology III score; SOFA, Sequential Organ Failure Assessment.
