# Supplementary material for: Relationship between baseline bicarbonate and 30-day mortality in patients with non-traumatic subarachnoid hemorrhage
Source: Front Neurol. 2024 Jan 3;14:1310327. doi: 10.3389/fneur.2023.1310327 (PMC10793108; doi:10.3389/fneur.2023.1310327)
Supplement: Supplementary file 5 [file Table_2.DOCX]

**Supplementary T2 Population characteristics between different groups.**

| **Variables** | **Group** | | | **p-value** |
| --- | --- | --- | --- | --- |
|  | **Total (n = 521)** | **Survivors (n = 398)** | **Non-survivors (n = 123)** |  |
| **Demographic** | | | | |
| Female, n (%) | 292 (56.0) | 226 (56.8) | 66 (53.7) | 0.542 |
| Age, years | 60.2 ± 14.4 | 58.4 ± 13.9 | 66.2 ± 14.5 | < 0.001 |
| Ethnicity, n (%) |  |  |  | < 0.001 |
| White | 326 (62.6) | 270 (67.8) | 56 (45.5) |  |
| Black | 46 ( 8.8) | 39 (9.8) | 7 (5.7) |  |
| Asian | 17 ( 3.3) | 10 (2.5) | 7 (5.7) |  |
| Other | 132 (25.3) | 79 (19.8) | 53 (43.1) |  |
| **Vital signs** | | | | |
| Heart rate, beats/min | 78.4 ± 13.7 | 76.9 ± 12.9 | 83.1 ± 14.9 | < 0.001 |
| MBP, mmHg | 81.9 ± 8.8 | 81.8 ± 8.9 | 82.2 ± 8.6 | 0.644 |
| RR, times/min | 18.0 ± 3.4 | 17.5 ± 3.0 | 19.7 ± 3.9 | < 0.001 |
| Temperature, ^◦^C | 37.0 ± 0.5 | 37.0 ± 0.4 | 36.9 ± 0.8 | 0.122 |
| SpO_2_, % | 97.8 (96.3, 99.0) | 97.6 (96.2, 98.9) | 98.4 (96.7, 99.4) | 0.011 |
| **Comorbidities, n (%)** | | | | |
| Myocardial infarction | 40 ( 7.7) | 28 (7) | 12 (9.8) | 0.322 |
| Congestive heart failure | 39 ( 7.5) | 28 (7) | 11 (8.9) | 0.482 |
| Chronic pulmonary disease | 77 (14.8) | 52 (13.1) | 25 (20.3) | 0.047 |
| Hypertension | 260 (49.9) | 196 (49.2) | 64 (52) | 0.589 |
| Diabetes | 70 (13.4) | 52 (13.1) | 18 (14.6) | 0.656 |
| Paraplegia | 61 (11.7) | 39 (9.8) | 22 (17.9) | 0.015 |
| Sepsis | 258 (49.5) | 183 (46) | 75 (61) | 0.004 |
| Renal disease | 27 ( 5.2) | 16 (4) | 11 (8.9) | 0.031 |
| Malignant cancer | 21 ( 4.0) | 14 (3.5) | 7 (5.7) | 0.297 |
| Severe liver disease | 6 ( 1.2) | 2 (0.5) | 4 (3.3) | 0.03 |
| Charlson comorbidity index | 4.0 (3.0, 6.0) | 4.0 (3.0, 6.0) | 6.0 (4.0, 7.0) | < 0.001 |
| **Laboratory results** | | | | |
| Glucose, mg/dl | 142.7 ± 36.5 | 136.9 ± 31.6 | 161.1 ± 44.7 | < 0.001 |
| RBC, 10^12^/L | 3.9 ± 0.7 | 4.0 ± 0.6 | 3.9 ± 0.8 | 0.199 |
| Hemoglobin, g/L | 11.9 ± 1.9 | 12.0 ± 1.8 | 11.4 ± 2.3 | 0.006 |
| Platelets, 10^9^/L | 215.7 ± 88.6 | 222.4 ± 85.1 | 193.6 ± 96.3 | 0.002 |
| WBC, 10^9^/L | 11.1 ± 4.8 | 10.7 ± 4.3 | 12.1 ± 6.2 | 0.008 |
| Sodium, mmol/L | 138.0 ± 3.9 | 138.1 ± 3.4 | 137.5 ± 5.2 | 0.133 |
| Calcium, mmol/L | 8.3 ± 0.7 | 8.4 ± 0.7 | 8.2 ± 0.8 | 0.011 |
| PT, s | 12.2 (11.3, 13.0) | 12.1 (11.3, 12.9) | 12.4 (11.4, 13.6) | 0.049 |
| APTT, s | 26.1 (23.6, 28.5) | 26.0 (23.6, 28.3) | 26.3 (23.7, 29.9) | 0.369 |
| Cr (mg/dL) | 0.7 (0.6, 0.9) | 0.7 (0.6, 0.9) | 0.8 (0.6, 1.1) | < 0.001 |
| BUN (mg/dL) | 12.0 (10.0, 17.0) | 11.0 (9.0, 15.0) | 16.0 (12.0, 23.5) | < 0.001 |
| Bicarbonate (Eq/L) | 23.4 ± 3.4 | 23.8 ± 3.2 | 22.1 ± 4.0 | < 0.001 |
| Quartiles of Bicarbonate, n (%) |  |  |  | < 0.001 |
| Q1(≤20 mEq/L) | 87 (16.7) | 52 (13.1) | 35 (28.5) |  |
| Q2(21-22 mEq/L) | 107 (20.5) | 73 (18.3) | 34 (27.6) |  |
| Q3(23-25 mEq/L) | 187 (35.9) | 157 (39.4) | 30 (24.4) |  |
| Q4(≥26 mEq/L) | 140 (26.9) | 116 (29.1) | 24 (19.5) |  |
| **Therapy and outcome, n (%)** | | | | |
| Endovascular therapy | 190 (36.5) | 161 (40.5) | 29 (23.6) | < 0.001 |
| Clipping of aneurysm | 37 ( 7.1) | 32 (8) | 5 (4.1) | 0.134 |
| Hydrocephalus | 154 (29.6) | 114 (28.6) | 40 (32.5) | 0.41 |
| **Scores** | | | | |
| GCS | 12.0 (7.0, 14.0) | 13.0 (8.0, 14.0) | 7.0 (3.0, 15.0) | < 0.001 |
| APSIII | 39.0 (27.0, 61.0) | 34.0 (25.0, 51.0) | 61.0 (44.5, 84.0) | < 0.001 |
| SOFA | 3.0 (2.0, 4.0) | 2.0 (2.0, 3.0) | 3.0 (2.0, 4.0) | 0.001 |

MBP, mean blood pressure; RR, respiratory rate; SpO2, percutaneous oxygen saturation;RBC, red blood cell; WBC, white blood cell; PT, prothrombin time; APTT, activated partial thromboplastin time; Cr, Creatinine; BUN, Blood urea nitrogen; GCS, Glasgow coma score; APSIII score, Acute Physiology III score; SOFA, Sequential Organ Failure Assessment.
