# Supplementary material for: Relationship between baseline bicarbonate and 30-day mortality in patients with non-traumatic subarachnoid hemorrhage
Source: Front Neurol. 2024 Jan 3;14:1310327. doi: 10.3389/fneur.2023.1310327 (PMC10793108; doi:10.3389/fneur.2023.1310327)
Supplement: Supplementary file 6 [file Table_3.DOCX]

**Supplementary T3 Univariate analysis**

| **Item** | **HR(95%CI)** | ***p***-value |
| --- | --- | --- |
| Sex |  |  |
| Male | Ref. |  |
| Female | 1.09 (0.76,1.55) | 0.637 |
| Age | 1.03 (1.02,1.05) | < 0.001 |
| Ethnicity |  |  |
| White | Ref. |  |
| Black | 0.91 (0.41,1.99) | 0.812 |
| Asian | 2.98 (1.36,6.55) | 0.006 |
| Other | 2.76 (1.9,4.02) | < 0.001 |
| Heart rate | 1.03 (1.02,1.04) | < 0.001 |
| MBP | 1.0055 (0.9858,1.0256) | 0.586 |
| RR | 1.15 (1.1,1.21) | < 0.001 |
| Temperature | 0.73 (0.53,1.01) | 0.058 |
| SpO_2_ | 0.9927 (0.9002,1.0948) | 0.884 |
| Myocardial infarction |  |  |
| No | Ref. |  |
| Yes | 1.3 (0.72,2.35) | 0.391 |
| Congestive heart failure |  |  |
| No | Ref. |  |
| Yes | 1.25 (0.67,2.32) | 0.482 |
| Chronic pulmonary disease |  |  |
| No | Ref. |  |
| Yes | 1.51 (0.98,2.35) | 0.064 |
| Hypertension |  |  |
| No | Ref. |  |
| Yes | 1.08 (0.76,1.54) | 0.653 |
| Diabetes |  |  |
| No | Ref. |  |
| Yes | 1.1 (0.67,1.81) | 0.717 |
| Paraplegia |  |  |
| No | Ref. |  |
| Yes | 1.7 (1.07,2.69) | 0.024 |
| Sepsis |  |  |
| No | Ref. |  |
| Yes | 1.64 (1.14,2.36) | 0.007 |
| Renal disease |  |  |
| No | Ref. |  |
| Yes | 1.84 (0.99,3.41) | 0.054 |
| Malignant cancer |  |  |
| No | Ref. |  |
| Yes | 1.32 (0.62,2.83) | 0.477 |
| Severe liver disease |  |  |
| No | Ref. |  |
| Yes | 3.88 (1.43,10.5) | 0.008 |
| Charlson comorbidity index | 1.18 (1.11,1.26) | < 0.001 |
| Glucose | 1.01 (1.01,1.02) | < 0.001 |
| RBC | 0.86 (0.65,1.13) | 0.272 |
| Hemoglobin | 0.89 (0.81,0.97) | 0.007 |
| Platelets | 0.9962 (0.9939,0.9985) | 0.001 |
| WBC | 1.05 (1.01,1.08) | 0.005 |
| Sodium | 0.97 (0.93,1.01) | 0.184 |
| Calcium | 0.74 (0.59,0.93) | 0.008 |
| PT | 1.04 (0.99,1.1) | 0.08 |
| APTT | 1.0065 (0.9901,1.0231) | 0.442 |
| Cr | 1.42 (1.23,1.64) | < 0.001 |
| BUN | 1.02 (1.02,1.03) | < 0.001 |
| Bicarbonate | 0.88 (0.83,0.92) | < 0.001 |
| Quartiles of Bicarbonate |  |  |
| Q1(≤20 mEq/L) | Ref. |  |
| Q2(21-22 mEq/L) | 0.7 (0.44,1.12) | 0.138 |
| Q3(23-25 mEq/L) | 0.32 (0.2,0.52) | < 0.001 |
| Q4(≥26 mEq/L) | 0.35 (0.21,0.58) | < 0.001 |
| Endovascular therapy |  |  |
| No | Ref. |  |
| Yes | 0.48 (0.32,0.73) | < 0.001 |
| Clipping of aneurysm |  |  |
| No | Ref. |  |
| Yes | 0.5 (0.2,1.22) | 0.129 |
| GCS | 0.89 (0.86,0.93) | < 0.001 |
| APSIII | 1.04 (1.03,1.04) | < 0.001 |
| SOFA | 1.16 (1.04,1.29) | 0.006 |
| Hydrocephalus |  |  |
| No | Ref. |  |
| Yes | 1.17 (0.8,1.7) | 0.42 |

MBP, mean blood pressure; RR, respiratory rate; SpO2, percutaneous oxygen saturation;RBC, red blood cell; WBC, white blood cell; PT, prothrombin time; APTT, activated partial thromboplastin time; Cr, Creatinine; BUN, Blood urea nitrogen; GCS, Glasgow coma score; APSIII score, Acute Physiology III score; SOFA, Sequential Organ Failure Assessment.
