# Supplementary material for: Relationship between baseline bicarbonate and 30-day mortality in patients with non-traumatic subarachnoid hemorrhage
Source: Front Neurol. 2024 Jan 3;14:1310327. doi: 10.3389/fneur.2023.1310327 (PMC10793108; doi:10.3389/fneur.2023.1310327)
Supplement: Supplementary file 7 [file Table_4.DOCX]

**Supplementary T4 Multivariate cox regression analyses for multiple mortality in non-traumatic SAH patients.**

| **Exposure** | **Non-adjust model** | | **Model I** | | **Model II** | |
| --- | --- | --- | --- | --- | --- | --- |
|  | **HR (95% CI)** | ***p*-value** | **HR (95% CI)** | ***p*-value** | **HR (95% CI)** | ***p*-value** |
| **24h mortality** | | | | | | |
| **Bicarbonate quartiles** |  |  |  |  |  |  |
| Q1(≤20 mEq/L) | 1 (Ref) |  | 1 (Ref) |  | 1 (Ref) |  |
| Q2(21-22 mEq/L) | 0.3 (0.12~0.77) | 0.012 | 0.29 (0.11~0.75) | 0.011 | 0.24 (0.08~0.7) | 0.009 |
| Q3(23-25 mEq/L) | 0.17 (0.07~0.44) | <0.001 | 0.17 (0.06~0.45) | <0.001 | 0.17 (0.06~0.48) | 0.001 |
| Q4(≥26 mEq/L) | 0.23 (0.09~0.59) | 0.002 | 0.24 (0.09~0.63) | 0.004 | 0.24 (0.08~0.7) | 0.009 |
| *p* for trend | 0.54 (0.39~0.76) | <0.001 | 0.55 (0.39~0.78) | 0.001 | 0.56 (0.38~0.83) | 0.004 |
| **Bicarbonate (per 1 increases)** | 0.82 (0.75~0.89) | <0.001 | 0.81 (0.74~0.89) | <0.001 | 0.83 (0.75~0.92) | <0.001 |
| **48h mortality** | | | | | | |
| **Bicarbonate quartiles** |  |  |  |  |  |  |
| Q1(≤20 mEq/L) | 1 (Ref) |  | 1 (Ref) |  | 1 (Ref) |  |
| Q2(21-22 mEq/L) | 0.4 (0.2~0.81) | 0.011 | 0.39 (0.19~0.79) | 0.009 | 0.45 (0.2~0.99) | 0.047 |
| Q3(23-25 mEq/L) | 0.15 (0.07~0.34) | <0.001 | 0.15 (0.07~0.34) | <0.001 | 0.2 (0.08~0.49) | <0.001 |
| Q4(≥26 mEq/L) | 0.23 (0.1~0.49) | <0.001 | 0.24 (0.11~0.55) | 0.001 | 0.35 (0.15~0.85) | 0.02 |
| *p* for trend | 0.53 (0.4~0.69) | <0.001 | 0.54 (0.41~0.71) | <0.001 | 0.63 (0.46~0.86) | 0.004 |
| **Bicarbonate (per 1 increases)** | 0.82 (0.77~0.88) | <0.001 | 0.82 (0.76~0.88) | <0.001 | 0.87 (0.8~0.95) | 0.001 |
| **7-day mortality** | | | | | | |
| **Bicarbonate quartiles** |  |  |  |  |  |  |
| Q1(≤20 mEq/L) | 1 (Ref) |  | 1 (Ref) |  | 1 (Ref) |  |
| Q2(21-22 mEq/L) | 0.56 (0.32~0.99) | 0.047 | 0.55 (0.31~0.97) | 0.04 | 0.73 (0.39~1.38) | 0.328 |
| Q3(23-25 mEq/L) | 0.2 (0.11~0.39) | <0.001 | 0.2 (0.1~0.39) | <0.001 | 0.3 (0.14~0.61) | 0.001 |
| Q4(≥26 mEq/L) | 0.22 (0.11~0.44) | <0.001 | 0.24 (0.12~0.49) | <0.001 | 0.38 (0.17~0.83) | 0.015 |
| *p* for trend | 0.54 (0.43~0.68) | <0.001 | 0.55 (0.43~0.69) | <0.001 | 0.66 (0.51~0.85) | 0.001 |
| **Bicarbonate (per 1 increases)** | 0.83 (0.78~0.88) | <0.001 | 0.83 (0.78~0.88) | <0.001 | 0.89 (0.83~0.95) | 0.001 |
| **In-hospital mortality** | | | | | | |
| **Bicarbonate quartiles** |  |  |  |  |  |  |
| Q1(≤20 mEq/L) | 1 (Ref) |  | 1 (Ref) |  | 1 (Ref) |  |
| Q2(21-22 mEq/L) | 0.69 (0.41~1.16) | 0.159 | 0.69 (0.41~1.16) | 0.157 | 0.95 (0.53~1.67) | 0.846 |
| Q3(23-25 mEq/L) | 0.34 (0.2~0.57) | <0.001 | 0.31 (0.18~0.54) | <0.001 | 0.5 (0.28~0.9) | 0.022 |
| Q4(≥26 mEq/L) | 0.39 (0.22~0.67) | 0.001 | 0.42 (0.24~0.74) | 0.003 | 0.68 (0.37~1.27) | 0.23 |
| *p* for trend | 0.68 (0.57~0.82) | <0.001 | 0.68 (0.57~0.83) | <0.001 | 0.83 (0.67~1.01) | 0.064 |
| **Bicarbonate (per 1 increases)** | 0.89 (0.84~0.93) | <0.001 | 0.88 (0.84~0.93) | <0.001 | 0.94 (0.89~1) | 0.045 |

Non-adjusted: no covariates were adjusted.

Model I: adjusted for age, sex, and ethnicity.

Model II: adjusted for age, sex, ethnicity, heart rate, RR, sepsis, Hemoglobin, Platelets, Charlson comorbidity index, Cr, endovascular therapy and GCS.

RR, respiratory rate; Cr, Creatinine; GCS, Glasgow coma score; HR, hazard ratio; CI, confidence interval; Ref, reference.
