# Supplementary material for: Relationship between baseline bicarbonate and 30-day mortality in patients with non-traumatic subarachnoid hemorrhage
Source: Front Neurol. 2024 Jan 3;14:1310327. doi: 10.3389/fneur.2023.1310327 (PMC10793108; doi:10.3389/fneur.2023.1310327)
Supplement: Supplementary file 8 [file Table_5.DOCX]

**Supplementary T5 Threshold effect analysis of the relationship of bicarbonate with 30-day mortality.**

| Outcome | HR (95% CI) | *p*-value |
| --- | --- | --- |
| Bicarbonate <25.67 | 0.831 (0.783,0.881) | < 0.001 |
| Bicarbonate ≥25.67 | 1.023 (0.803,1.304) | 0.8515 |
| Likelihood Ratio test |  | 0.048 |

The age, sex, and ethnicity are adjusted. HR, hazard ratio; CI, confidence interval.
